# The Brazilian Portuguese Version of the Behavioral Regulation in Exercise Questionnaire 3 (BREQ-3) Is Reliable and Valid for Assessing Motivational Regulations and Self-Determination in Exercise Among Adults Aged 50 Years or Older: A Methodological Study

**DOI:** 10.3390/ijerph22010082

**Published:** 2025-01-09

**Authors:** Jacyara de Oliveira Vanini, Manuela Karloh, Ricardo Coelho Bosco, Michelle Gonçalves de Souza, Marlus Karsten, Darlan Laurício Matte

**Affiliations:** 1 Graduate Program in Physical Therapy, Santa Catarina State University, Florianópolis 88080-350, Brazil; jacyvanini@outlook.com (J.d.O.V.); manuela.karloh@udesc.br (M.K.); ricardobosco@hotmail.com (R.C.B.); marlus.karsten@udesc.br (M.K.); 2 Archdiocesan Consul Carlos Renaux Hospital, Brusque 88353-902, Brazil; 3 Department of Physical Therapy, Santa Catarina State University, Florianópolis 88080-350, Brazil; 4 Municipal City Hall of Garopaba, Garopaba 88495-000, Brazil; 5 Santa Catarina State Health Department, Florianópolis 88015-270, Brazil; mgs.michellegoncalves@gmail.com

**Keywords:** aging, motivation, patient-reported outcome measures, outcome assessment, frailty

## Abstract

The study aimed to investigate the reliability, construct, and discriminant validity of the Behavioral Regulation in Exercise Questionnaire 3 (BREQ-3) for evaluating motivational regulations and self-determination for exercise in Brazilian adults aged 50 years or older. The study assessed motivation for exercise, peripheral muscle strength, physical performance, functional capacity, cardiovascular fitness, and frailty phenotype. Two raters independently applied the BREQ-3. The reliability was evaluated using internal consistency, test–retest reliability, and agreement. Construct validity was tested with Spearman’s correlation coefficient and discriminant validity with the Kruskal–Wallis test. Eighty individuals participated in the reliability study, and 136 participated in the validation study. Motivational regulation and Self-Determination Index (SDI) internal consistency ranged from 0.55 to 0.82. Test–retest reliability ranged from 0.77 (95% CI 0.64–0.85) to 0.91 (95% CI 0.85–0.94), and inter-rater ranged from 0.80 (95% CI 0.68–0.87) to 0.92 (95% CI 0.88–0.95), except for amotivation, which had poor inter-rater and test–retest reliability. Additionally, moderate to weak correlations between various types of motivation and physical function tests were found (*p* < 0.05). Frail and pre-frail participants had lower SDI, integrated, and intrinsic motivation regulation than non-frail individuals (*p* < 0.05). This study confirmed the reliability, construct, and discriminative validity of the Brazilian Portuguese version of the BREQ-3 for evaluating motivational regulations and self-determination for exercise in older adults.

## 1. Introduction

Participation in physical activities among older adults is a recognized approach to reducing the risk of chronic diseases and mortality while improving overall health and quality of life [1]. However, physical inactivity remains highly prevalent in this age group, with recent data showing a decline in activity levels with age [2]. A systematic review by Franco and colleagues [3] highlighted that lack of motivation is one of the main barriers preventing older adults from engaging in exercise, even when they acknowledge its benefits. Nevertheless, initiating or maintaining regular exercise programs remains a significant challenge [4].

Given the difficulty of promoting exercise-related behavior change, Self-Determination Theory (SDT) has been widely used to study these processes [5,6,7]. SDT describes the role of motivation in the process of behavior change. This theory defines motivation as a multifaceted concept composed of three distinct types of motivation that vary from less to more self-determined: amotivation, controlled motivation, and autonomous motivation. Each type of motivation has motivational regulations that reflect the mechanisms that determine the quality of motivation concerning a specific activity. In addition, motivational regulations are affected by the degree to which the social context meets their basic psychological needs, which include autonomy, competence, and relatedness. Understanding the different motivational regulations is essential in the various contexts that manipulate physical exercise. Each type of motivation can produce different results and influence the adoption and maintenance of regular physical exercise practice [7,8].

In this context, several instruments based on SDT have been developed to measure human motivation. One of these instruments is the Behavioral Regulation in Exercise Questionnaire (BREQ), created to quantify motivational regulations related to physical exercise practice [9]. The BREQ was revised and expanded by Markland and Tobin [10], creating the BREQ-2, which was later modified to evaluate the entire continuum of motivational regulations proposed by SDT, resulting in the BREQ-3 [10,11]. The BREQ-3 consists of 23 items and quantifies the motivational regulations of the self-determination continuum: amotivation, external, introjected, identified, integrated, and intrinsic regulations [10,11]. In Brazil, it was translated and validated for adult participants of fitness clubs aged between 18 and 54 years [12]. However, according to the authors, only 8% of their sample was composed of adults older than 41 years old.

These characteristics from Guedes and Sofiati’s study [12] raise questions about the applicability of the BREQ-3 for assessing exercise motivation in older adults who do not engage in fitness club activities in Brazil. The context in which studies are conducted can influence the motivational profile and reasons for engaging in physical exercise [12]. Older adults often face unique challenges related to motivation, such as physical limitations, health literacy [13], cognitive decline, and changing social contexts [14,15,16,17], which may affect their responses to self-report measures like the BREQ-3.

Research on physical activity barriers and motivators in this age group highlights several key factors influencing participation, including health concerns, fear of injury or pain, self-motivation, social support, confidence, and access to assistance [15,17]. These factors, along with life circumstances and shifting priorities, can lead to distinct interpretations of motivational items among older adults compared to younger age groups. As a result, it is essential to categorize older adults into specific age ranges to accurately assess how they conceptualize motivation for physical activity [18].

To address these gaps, a study focusing on adults aged 50 years or older is needed to ensure that the BREQ-3 adequately captures the different forms of motivation for physical activity in this group. By examining motivational regulations in older adults, researchers can develop more effective strategies to promote physical activity, improve health outcomes, and enhance quality of life. Ensuring the tool’s reliability, validity, and relevance for aging populations will provide meaningful insights and support interventions tailored to this group.

Given the above, the present study aimed to investigate the reliability and validity of the BREQ-3 for evaluating motivational regulations for exercise in adults aged 50 years or older in Brazil.

## 2. Materials and Methods

### 2.1. Study Protocol

This methodological study evaluates the reliability, construct validity, and discriminant validity. The study is part of an umbrella project titled ‘Physical Activity Level, Sedentary Behavior, Motivation for Physical Exercise, Frailty, and Sarcopenia in Elderly Users of the Unified Health System (ATIVO-SUS)’ and was approved by the Ethics and Research Committee on Human Beings at the Santa Catarina State University (CAAE: 40631920.5.0000.0118). All participants read and signed an informed consent form. The sample was selected using non-probabilistic methods and included participants of both sexes, aged over 50, who engage in exercise or physical activity as part of rehabilitation services in the public sectors of São João Batista and Garopaba, Santa Catarina, Brazil. The exclusion criteria were neuromuscular or osteoarticular diseases that limit participation in the evaluation procedures, wheelchair users, and those with cognitive deficits (Mini-Mental State Examination < 17 points) [19].

Data collection took place between September 2021 and March 2022. with individuals participating in exercise or physical activity as part of rehabilitation services in the public health sectors of São João Batista and Garopaba, Brazil. Assessments were conducted in designated rooms within the Physiotherapy Services of the Public Health System. All evaluations were carried out by trained researchers, ensuring a standardized protocol for the physical tests and questionnaires.

This study was divided into two arms. Arm one comprised the reliability study, and arm two the validation study. Data collection from the reliability study occurred over two days and was conducted by two independent evaluators (R1—RCB and R2—AFCA). They administered the BREQ-3 as an interview. On day one, the BREQ-3 was administered by R1 and, 30 min later (5-min tolerance), by R2. Participants also underwent anamnesis, anthropometric measurements, and assessments of peripheral muscle strength, physical performance, and functional capacity. On day two, after a 12- to 20-day interval, participants answered the BREQ-3 again (administered by R1) and performed cardiovascular fitness and frailty phenotype assessments. Individuals completed the same protocol for the validation study, except that the BREQ-3 was administered only once.

Motivational regulation assessment: The BREQ-3 was developed by Markland and Tobin [10] and Wilson et al. [11] and later translated and validated into Brazilian Portuguese [12]. It consists of 23 statements that begin with the question “Why do you engage in physical exercise?” followed by a five-point Likert scale for the participant to evaluate their level of agreement. The values range from 0 (“not at all true”) to 4 (“totally true”). The six motivational regulations of the self-determination continuum (amotivation, external regulation, introjected regulation, identified regulation, integrated regulation, and intrinsic regulation) are calculated as the mean score of the items corresponding to each regulation. Also, the self-determination index (SDI) was calculated using the following equation: SDI = (−3 × amotivation) + (−2 × external regulation) + (−1 × introjected regulation) + (1 × identified regulation) + (2 × integrated regulation) + (3 × intrinsic regulation). The SDI ranges from −24 to +24. The higher the score, the more the individual recognizes self-determined reasons for exercising [10,11].

Other assessments: Weight and height were measured to record the participant’s body mass index (BMI). The weight was determined using a digital scale manufactured by Mondial (model Smart Black BL-05), with the participants wearing light clothing and no shoes. The height, on the other hand, was measured using a Portable Personal Caprice Sanny Stadiometer, with the participants standing upright and barefoot [20].

To assess peripheral muscle strength, a handgrip test was conducted with a Hand Dynamometer Type Smedley (SAEHAN, model SH5002). The data collection procedures adhered to the standardized grip strength testing guidelines established by the American Society of Hand Therapists. Three attempts were made with the participant’s dominant hand, and the average was recorded [21,22]. The result was classified according to Fried et al. [23].

The Short Physical Performance Battery (SPPB) assessed physical performance through three tests: (1) standing balance performed in three positions, (2) lower extremity strength and power measured by five repetitions sit-to-stand from a chair, and (3) gait speed. Each test was scored from 0 (inability to perform the task) to 4 (best performance) [24]. Also, the time to complete tasks 2 and 3 was registered, and the average gait speed was calculated for task 3 (m/s) [25]. The SPPB total score ranges from 0 (worst performance) to 12 (best performance), with performance categorized as 0–3 (disability/very poor performance), 4–6 (poor performance), 7–9 (moderate performance), and 10–12 (good performance) [26].

The 4-m gait speed test (4mGST) was used to assess functional capacity. Participants were instructed to walk four meters at their usual pace. The time to complete the distance was recorded, and the average gait speed was calculated (m/s). Higher gait speed indicates better functional capacity [27].

Cardiovascular fitness was evaluated using The Fitness Test from the Polar M430 heart rate monitor. It estimates an individual’s maximal oxygen consumption after a minimum of 20 min of rest in the supine position. The monitor was placed on the participant’s wrist, and the sensor was attached to the skin [28].

The frailty phenotypes were assessed through five components: unintentional weight loss, low level of physical activity, fatigue, muscular weakness, and slow gait speed. Self-reported instruments assessed weight loss (>4.5 kg or 5% of body weight in the previous year), fatigue, and physical activity level. The other two items were assessed through the handgrip and 4mGST mentioned above. An individual was considered frail if they presented three or more of the five components, pre-frail if they scored one or two, and non-frail if they did not score any of the components [23,29].

### 2.2. Statistical Analysis

The data analysis was performed using IBM SPSS Statistics^®^ version 20.0 (IBM Corporation, Armonk, NY, USA). GraphPad Prism version 5.0 (GraphPad Inc., San Diego, CA, USA) was used to create figures. Internal consistency was assessed using Cronbach’s alpha coefficient. The reliability of the data was evaluated using the intraclass correlation coefficient (ICC) according to the classification by Fleiss et al. [30]. To verify the limits of agreement between inter-rater (R1 vs. R2) and test–retest (R1, assessment one vs. R1, assessment two) applications, Bland–Altman plots were used. The standard error of measurement (SEM) was calculated using the formula SEM = SD × √(1-ICC), where SD represents the standard deviation of the scale scores administered by R1. The minimum detectable change (MDC95%) was calculated using the equation MDC95% = 1.96 × √2·SEM [31]. Spearman’s correlation coefficient was used to evaluate the construct validity of BREQ-3, considering peripheral muscle strength, physical performance (tests 2 and 3 of SBBP), functional capacity, and cardiovascular fitness. Discriminant validity was tested by comparing the motivational regulations and SDI among frailty phenotypes using the Kruskal–Wallis followed by Mann–Whitney U tests. A significance level of 5% was adopted. For the reliability study, the sample size was estimated on the basis of the recommendation of an adequate sample size for measurement error and the reliability and internal consistency studies of the Consensus-based Standards for the Selection of Health Status Measurement Instruments (COSMIN) of 50–99 patients [32]. For the validity study, with an expected correlation of 0.30, a bilateral alpha of 0.05, a power of 0.10, and a drop-out rate of 20%, 136 patients were estimated [33]. Also, COSMIN recommends a sample of at least 100 patients for construct validity studies using other outcome measurement instruments [32].

## 3. Results

Eighty individuals participated in arm 1, the reliability study, while 136 individuals participated in arm 2, the validation study. The validation study’s sample comprised 80 individuals from the reliability study plus 56 individuals from a second study center. Table 1 presents the sample characteristics.

The motivational regulation’s internal consistency ranged from 0.55 to 0.82, as shown in Table 2. The SDI’s internal consistency was 0.75. Inter-rater reliability was good for external, introjected, identified, and integrated regulations and SDI. Intrinsic regulation showed excellent inter-rater reliability. Test–retest reliability was good for external, introjected, identified, and SDI regulations and excellent for integrated and identified regulations. However, amotivation had poor inter-rater and test–retest reliability. Table 2 also presents the SEM and MDC95% for motivational regulations and SDI.

**Figure 1:**
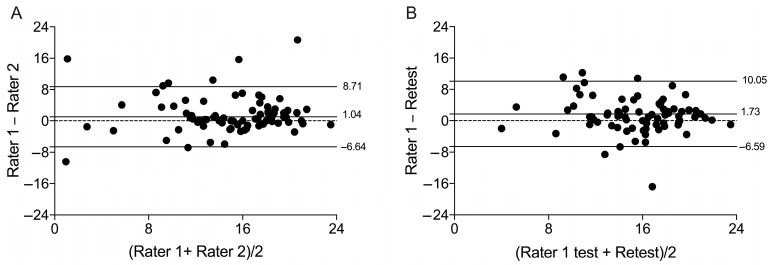
Bland–Altman plots for the inter-rater reliability (panel **A**) and test–retest reliability (panel **B**) of the Self-Determination Index (SDI) from the Brazilian version of the Behavior Regulation in Exercise Questionnaire 3 (BREQ-3). Note: n = 80; fewer visible dots indicate overlapping data points (different participants scored identically).

**Figure 2:**
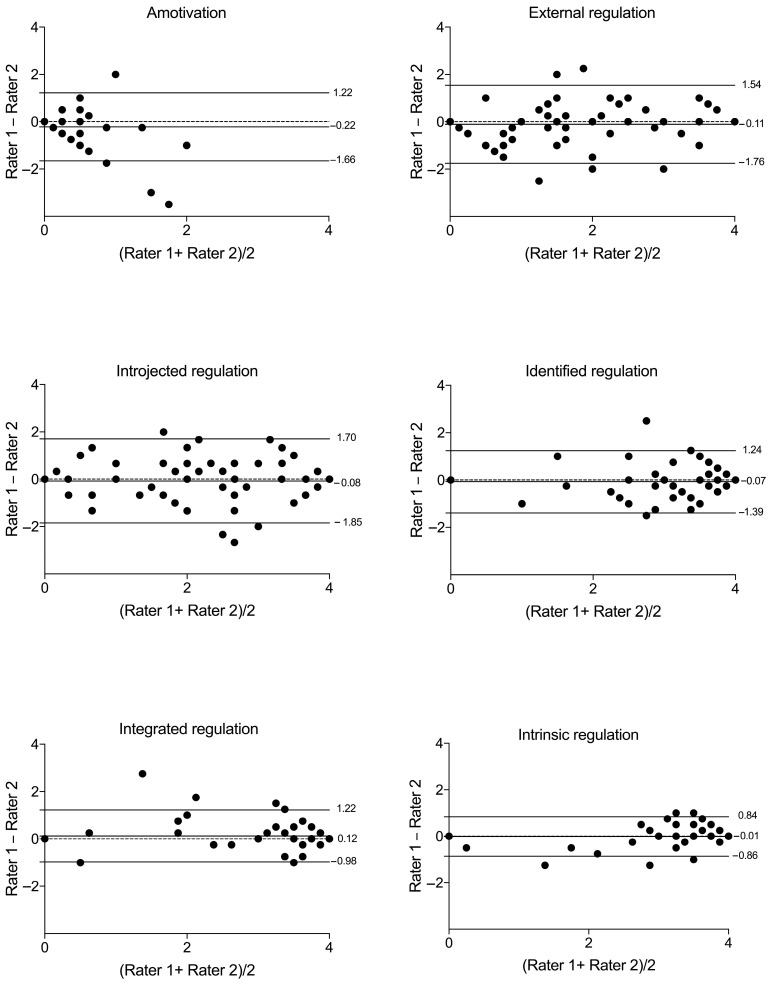
Bland–Altman plots for the inter-rater agreement between assessments of each motivational regulation assessed with the Brazilian version of the Behavior Regulation in Exercise Questionnaire 3 (BREQ-3). Note: n = 80; fewer visible dots indicate overlapping data points (different participants scored identically).

**Figure 3:**
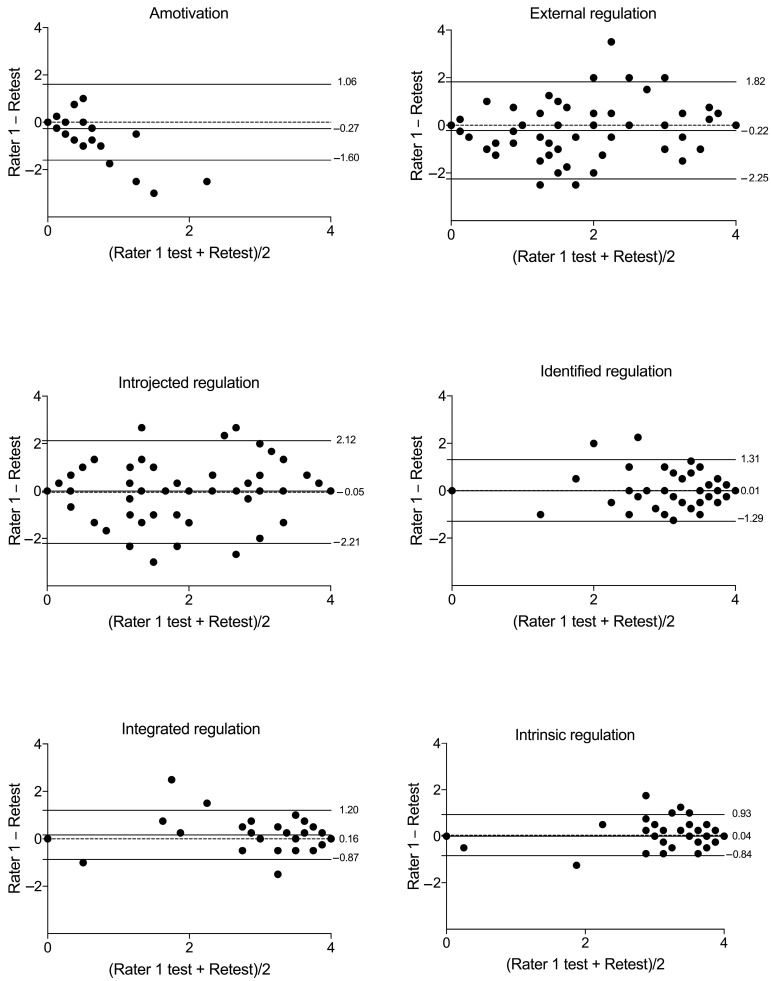
Bland–Altman plots for the test–retest agreement between assessments of each motivational regulation assessed with the Brazilian version of the Behavior Regulation in Exercise Questionnaire 3 (BREQ-3). Note: n = 80; fewer visible dots indicate overlapping data points (different participants scored identically).

The mean difference in SDI between the two measurements was 1.04 units for inter-rater assessment, with limits of agreement ranging from −6.64 to 8.71. For the test–retest, the mean difference was 1.73, with limits of agreement ranging from −6.59 to 10.05. In the inter-rater analysis, 7.5% of total data points fell outside the limits of agreement, while in the test–retest analysis, 6.2% did. Figure 1 illustrates these findings. Bland–Altman plots demonstrated minor differences between the applications’ agreement for each motivational regulation individually, with most applications falling within the agreement limits, as shown in Figure 2 and Figure 3.

Table 3 displays the inter-correlations between BREQ-3 subscales. Subscales adjacent to the self-determination continuum exhibit higher and more positive correlations when compared to subscales distant from one another on the continuum, which are negatively correlated.

The construct validity was demonstrated by a moderate to weak correlation between various types of motivation with physical function tests. External regulation was found to be correlated with sit-to-stand (r = 0.37, *p* < 0.01), while introjected regulation was correlated with both sit-to-stand (r = 0.46, *p* < 0.01) and 4mGST (r = −0.36, *p* < 0.01). Integrated regulation was also found to be correlated with sit-to-stand (r = 0.30, *p* < 0.01) and 4mGST (r = −0.34, *p* < 0.01), while intrinsic regulation was correlated with both sit-to-stand and 4mGST (r = −0.30, *p* < 0.01).

Regarding discriminant validity, motivational regulations and SDI varied according to frailty phenotypes. Frail and pre-frail participants had lower SDI, integrated, and intrinsic motivation regulations; pre-frail had lower introjected regulation than non-frail individuals (*p* < 0.01). However, the motivational profile did not differ between pre-frail and frail individuals (*p* > 0.05) (Table 4).

## 4. Discussion

The present study assessed the reliability, construct, and discriminant validity of the Brazilian Portuguese version of the BREQ-3 in adults aged 50 years or older. The study’s results confirmed that the BREQ-3 is a reliable and valid instrument for evaluating this population’s motivational regulation and self-determination related to exercise.

The BREQ-3 was developed to solve the conceptual disagreement between the BREQ-2 and SDT. Although the BREQ-2 is the most widely used and validated tool for measuring SDT motivation in exercise or physical activity [34,35,36,37], it only addresses three out of the four types of extrinsic motivation: external regulation, introjected regulation, and identified regulation [10]. Furthermore, it does not include integrated regulation due to its similarity to intrinsic regulation. Even if both integrated and intrinsic regulations involve a sense of volition, integrated regulation is still influenced by external factors, while intrinsic regulation is solely driven by internal pleasure [7,8]. To address this issue, Wilson et al. [11] developed four items to measure integrated regulation and ensure that the entire SDT’s motivational continuum in exercise is captured. These items were then incorporated into the BREQ-2, resulting in the development of the BREQ-3 [10,11]. Since its creation, the BREQ-3 has been validated in Spanish [36], Portuguese [34], Chinese [35], Italian [37], and Brazilian Portuguese [12].

Our study’s results indicated that the internal consistency of SDI and motivational regulations was acceptable, except for identified regulation. While it is generally suggested that Cronbach’s alpha should be at least 0.70 for satisfactory reliability, an absolute value of 0.60 is also acceptable for subscales containing four items [38], as is the case of motivational regulation subscales in the BREQ-3. Our findings are consistent with previous studies [12,36,39,40,41], particularly those conducted with non-young exercise participants [39,40,41]. For example, a study of 118 individuals with schizophrenia with an average age of 44.53 ± 9.74 years reported internal consistency ranging from 0.59 to 0.81, with identified regulation having the lowest value [39]. Additionally, in the first Brazilian Portuguese validation of BREQ-3, conducted with an adult exercising sample aged 18 to 54, identified regulation was the only subscale with a Cronbach’s alpha value below 0.70 [12]. Identified regulation is considered the most autonomous form of extrinsic motivation, where the behavior is voluntarily engaged in and perceived as valuable and meaningful, even though it still serves as a means to achieve an objective (e.g., the individual values the benefits of exercise and finds it important) [6,42,43].

Recent studies have investigated alternative structural equation models of the BREQ-3 using different analytical approaches, such as exploratory structural equation modeling, bi-factor confirmatory factor analysis, and bi-factor exploratory structural equation modeling, in addition to the typical confirmatory factor analysis [35]. These studies have found that BREQ-3’s item 19 had an unusually high factor loading compared to the other items in the identified regulation subscale in all the models. In the bi-factor exploratory structural equation modeling, item 19 had a significantly low loading on the autonomous motivation factor and a significantly high loading on the controlled motivation factor. These results suggest that participants may perceive item 19 as more controlling than an autonomous measure of exercise motivation [35].

It is worth noting that previous studies have also identified issues regarding item 19, which corresponded to item 17 in BREQ-2 when investigating the validity of BREQ-2 [44,45] and BREQ-3 [36]. As a result, some authors have eliminated item 19 to improve the overall model fit in the Chinese [44] and Spanish versions [45] of the BREQ-3.

Our study’s findings indicated that the BREQ-3 is reliable for inter-rater and test–retest assessments, except for amotivation. This is the first time that this type of reliability has been tested for the BREQ-3. Previous BREQ-3 validation studies involved participants completing the questionnaire independently. Before our study, the use of interviews for administering the BREQ-2 had been reported in the validation of the Brazilian Portuguese version of BREQ-2 in chronic respiratory disease patients [46] and in the Chinese version in nursing home residents [41]. However, we chose to apply the BREQ-3 through interviews for three reasons: (1) to minimize missing data; (2) to improve understanding of the items in older adults (by listening instead of reading); and (3) to support the use of BREQ-3 in clinical or rehabilitation exercise settings, where patient-related outcome measurements are typically conducted through interviews. Therefore, our results suggest that the BREQ-3 is a suitable instrument for clinical practice, where different staff members can perform assessments of individuals on different days. Furthermore, the MDC95% for each motivational regulation and SDI can aid in interpreting interventional results. Changes above the MDC95% are deemed to be real [47].

As this is the first study to administer the BREQ-3 through an interview format between the rater and the patient, the absence of intraclass correlation coefficients from other populations or settings is a limitation. However, as the amotivation subscale is the same in the BREQ-2 and BREQ-3 (BREQ-3 comprises BREQ-2 plus the additional integrated regulation items), we could compare our findings with ICCs from BREQ-2 validation studies, which reported acceptable reliability values [41,46,48]. Our study’s poor reliability for amotivation may have occurred because the entire sample scored within the initial range of the scale (0 to 2 points out of the 0 to 4 scale range) in the interrater analysis, and all but one participant scored 0 to 2 points in the test–retest analysis. It is known that reliability may vary at the very low or very high ranges of detection [49]. Thus, we believe the lack of reliability regarding amotivation was an isolated finding in our study, as this subscale behaved similarly to the other five motivational regulations in other studies.

The study’s results indicated that the different types of motivation exhibited a simplex structure, where items had correlations as advocated by the theoretical framework of SDT. This structure demonstrated positive correlations between regulation types closer on the continuum and less positive or negative correlations between those farther away [6,8]. These findings agree with previous validation studies [12,34,36,39,48,50,51] and provide strong evidence for the Self-Determination continuum, a fundamental aspect of the organismic integration sub-theory of SDT [43]. In Brazilian Portuguese-speaking older adults, the BREQ-3 effectively measured the various types of behavioral regulations, ranging from the least self-determined (amotivation) to the most self-determined (intrinsic motivation). These results further support the validity of the BREQ-3 and the conceptualization of the Self-Determination continuum in this population.

This is the first study to test and provide initial evidence supporting the construct validity of BREQ-3 scores among older adults using other constructs of functional status. Our results suggest that the least self-determined, or in other words, controlled forms of motivation, correlated with greater magnitude with the worst performance in functional tests. The literature has extensively reported the relationship between motivation regulations and health outcomes [7]. We selected physical performance and functional capacity measures for the validation process, as these outcomes are directly linked to exercise and better reflect the behavior of interest in our research. Since the BREQ-3 assesses exercise-related motivation, it was essential to use exercise-specific outcomes such as functional capacity and performance, which are closely tied to physical activity.

The findings that showed the discriminant validity of the BREQ-3 was another strength of this study. The less autonomous motivated, and self-determined motivational profile of frail and pre-frail participants compared to non-frail participants indicates that these individuals may face challenges in adhering to and maintaining exercise behavior [8], emphasizing the need for exercise and rehabilitation programs to create a supportive and autonomous environment to fulfill their basic psychological needs [52], irrespective of frailty severity.

A limitation of our study is that no other instruments are available in Brazilian Portuguese to evaluate motivational regulation in exercise for general or disease-specific populations. Therefore, criterion validity could not be assessed, which should be addressed in future research. Another limitation is that our sample did not score amotivation across the entire range of the subscale punctuation, which could have impacted its reliability analysis [49]. Thus, further investigation could be conducted to clarify our findings on the reliability of amotivation since they do not align with previous evidence [41,46], including a study conducted on Brazilian older adults with chronic respiratory disease using the BREQ-2 [46]. Additionally, our study did not account for gender differences or prior motor experience, which could have influenced the results. Previous motor experience, in particular, could act as a facilitator or a limitation depending on the individual’s background. However, we did not conduct any subgroup analysis based on these factors, which is a point that should be addressed in future research. Lastly, data collection occurred during the COVID-19 pandemic, and patient-reported outcomes, particularly behavior-related ones such as motivational regulation, could have been influenced by the restrictions imposed by COVID-19 [53]. Nonetheless, our analyses agree with previously reported findings in other languages, age groups, and contexts.

## 5. Conclusions

In conclusion, our study found that the Brazilian Portuguese version of the Behavioral Regulation in Exercise Questionnaire 3 (BREQ-3) is reliable and valid for assessing motivational regulation and self-determination related to exercise in adults aged 50 years or older.

## Figures and Tables

**Table 1:** Sample characteristics.

Variable	Reliability Study (n = 80)	Validation Study (n = 136)
Age, years	62.8 ± 8.0	65.5 ± 8.0
Sex *		
Female	58 (72.5)	104 (76.5)
Male	22 (27.5)	32 (23.5)
Body mass, kg	70.7 ± 13.8	70.1 ± 12.3
Height, m	1.60 ± 0.92	1.60 ± 0.93
BMI, kg/m^2^	27.4 ± 4.16	27.3 ± 3.42
Handgrip Strength, kgf	24.9 ± 8.6	25.3 ± 7.08
Handgrip weakness		
Yes	26 (32.5)	34 (25)
No	54 (67.5)	102 (75)
SPPB *		
Very poor	3 (3.8)	4 (2.9)
Poor	22 (27.5)	41 (30.1)
Moderate	55 (68.8)	91 (66.9)
Good	-	-
4-Meter Gait Speed Test, s	3.29 ± 0.99	3.17 ± 0.93
4-Meter Gait Speed Test, m/s	0.82 ± 0.24	0.79 ± 0.23
Cardiovascular fitness	25.6 ± 6.29	28.7 ± 6.96
Frailty		
Non-frail	24 (30)	24 (17.6)
Pre-frail	43 (53.8)	92 (67.6)
Frail	13 (16.4)	20 (14.7)

Data presented as mean ± standard deviation, except when indicated. * Data presented in absolute and relative frequency; BMI: body mass index; SBBP: Short Physical Performance Battery; kg: kilogram; m: meters; kg/m^2^: kilograms per square meter; pts: points; kgf: kilogram-force; m/s: meters per second.

**Table 2:** Motivational regulation and Self-Determination Index scores for each application, and reliability of the Brazilian version of the Behavior Regulation in Exercise Questionnaire 3 (BREQ-3).

BREQ-3	Evaluator 1 Mean ± SD	Evaluator 1 Retest Mean ± SD	Evaluator 2 Mean ± SD	Test-Retest ICC (95% CI)	Inter-Rater ICC (95% CI)	Cronbach’s α	SEM	MDC95%
Amotivation	0.28 ± 0.57	0.15 ± 0.32	0.17 ± 0.38	0.25 (−0.12–0.50)	0.25 (−0.14–0.51)	0.61	0.59	1.63
External regulation	1.47 ± 0.91	1.33 ± 1.01	1.40 ± 1.06	0.77 (0.64–0.85)	0.87 (0.80–0.92)	0.60	0.53	1.47
Introjected regulation	1.59 ± 1.47	1.54 ± 1.55	1.61 ± 1.40	0.83 (0.74–0.89)	0.88 (0.81–0.92)	0.72	0.56	1.54
Identified regulation	3.36 ± 0.64	3.36 ± 0.68	3.27 ± 0.72	0.79 (0.68–0.87)	0.80 (0.68–0.87)	0.55	0.35	0.98
Integrated regulation	2.79 ± 1.12	2.90 ± 1.13	2.92 ± 1.10	0.91 (0.85–0.94)	0.89 (0.83–0.93)	0.82	0.34	0.80
Intrinsic regulation	3.30 ± 0.75	3.32 ± 0.79	3.27 ± 0.84	0.90 (0.85–0.94)	0.92 (0.88–0.95)	0.77	0.32	0.64
SDI	13.43 ± 4.86	14.43 ± 4.48	14.00 ± 5.07	0.82 (0.68–0.89)	0.88 (0.81–0.93)	0.75	2.55	7.06

Data presented as mean ± standard deviation, except when indicated. Data presented in absolute and relative frequency; BREQ-3: Behavioral Regulation in Exercise Questionnaire 3; SD: standard deviation; ICC: intraclass correlation coefficient; CI: confidence interval; Cronbach’s α: Cronbach’s alpha coefficient; SEM: standard error of measurement; MDC95%: minimum detectable change at the 95% confidence level; SDI: Self-Determination Index.

**Table 3:** Correlations among BREQ-3 subscales.

	1	2	3	4	5	6	SDI
1. Amotivation	1	0.16	−0.08	−0.22 *	−0.38 *	−0.18	−0.59 **
2. External	-	1	0.37 *	0.11	0.08	0.01	−0.64 **
3. Introjected	-	-	1	0.41 **	0.37 **	0.40 **	−0.20
4. Identified	-	-	-	1	0.39 **	0.45 **	0.29 *
5. Integrated	-	-	-	-	1	0.51 **	0.47 **
6. Intrinsic	-	-	-	-	-	1	0.42 **
SDI	-	-	-	-	-	-	1

1. Amotivation; 2. External regulation; 3. Introjected regulation; 4. Identified regulation; 5. Integrated regulation; 6. Intrinsic regulation; SDI: Self-Determination Index. * *p* < 0.05; ** *p* < 0.01.

**Table 4:** Discriminant validity of the motivational regulations and Self-Determination Index (SDI) from Behavioral Regulation in Exercise Questionnaire 3 (BREQ-3) according to frailty phenotypes.

	Non-Frail (n = 23)	Pre-Frail (n = 92)	Frail (n = 18)	*p*
Amotivation	0.21 ± 0.48	0.28 ± 0.53	0.35 ± 0.82	0.55
External	1.48 ± 0.98	1.45 ± 0.87	1.57 ± 1.00	0.71
Introjected	2.25 ± 1.21 *	1.39 ± 1.41	2.04 ± 1.74	0.04
Identified	3.53 ± 0.51	3.34 ± 0.65	3.30 ± 0.73	0.41
Integrated	3.68 ± 0.58 *†	2.60 ± 1.11	2.69 ± 1.20	<0.01
Intrinsic	3.72 ± 0.38 *†	3.18 ± 0.81	3.33 ± 0.63	<0.01
SDI	16.2 ± 4.38 *†	12.9 ± 4.54	12.5 ± 5.96	<0.01

*p*: significance level. External: external regulation; Introjected: introjected regulation; Identified: identified regulation; Integrated: integrated regulation; Intrinsic: intrinsic regulation; SDI: Self-Determination Index. * *p* < 0.05 vs. pre-frail; † *p* < 0.05 vs. frail.

## Data Availability

The data presented in this study are available upon request from the corresponding author due to confidentiality issues determined by the Ethics Committee, but they are available from the principal investigator upon reasonable request and provided they are not used for commercial purposes.
